# Can Artificial Intelligence Match Human Expertise in Long‐Term Periodontal Prognosis? A Comparative Accuracy Study

**DOI:** 10.1111/jcpe.70191

**Published:** 2026-08-21

**Authors:** Pablo Antonio López‐Galindo, Giuseppe Troiano, Matteo Serroni, Debora R. Dias, Pasquale Santamaría, Luigi Nibali, Gustavo Avila‐Ortiz, Andrea Ravidà

**Affiliations:** ^1^ Department of Periodontics and Preventive Dentistry University of Pittsburgh Pittsburgh Pennsylvania USA; ^2^ Department of Medicine and Surgery LUM University Casamassima Italy; ^3^ Periodontology Unit, Department of Innovative Technologies in Medicine & Dentistry University ‘G. D'Annunzio’ of Chieti‐Pescara Chieti Italy; ^4^ Department of Dentistry State University of Maringa Maringa Brazil; ^5^ Periodontology Unit, Centre for Host‐Microbiome Interactions, Faculty of Dentistry, Oral and Craniofacial Sciences King's College London London UK; ^6^ Department of Oral Medicine, Infection, and Immunity Harvard School of Dental Medicine, Harvard University Boston Massachusetts USA

**Keywords:** artificial intelligence, clinical decision support system, periodontitis, prognosis, tooth loss

## Abstract

**Aim:**

To compare the prognostic performance of an artificial intelligence (AI) model with that of experienced clinicians in predicting tooth loss over a 10‐year period.

**Materials and Methods:**

An AI model trained on structured clinical and radiographic data was compared with 12 periodontists and 11 general dentists (GDs), who independently assigned prognostic scores (0–10 scale) to 300 teeth with known 10‐year outcomes. AI and clinician performance were evaluated under a fixed threshold and group‐specific optimal thresholds. Accuracy, sensitivity, specificity, predictive values, area under the receiver operating characteristic curve and calibration were used for comparison.

**Results:**

Using the fixed threshold (score > 5 = survival), clinicians achieved higher overall accuracy than AI (75.6% periodontists, 74.9% GDs, 69.2% AI; *p* < 0.05), with sensitivity low and comparable across groups (14.7%–22.7%). Using group‐specific optimal thresholds, AI achieved the highest accuracy for all‐cause tooth loss (67.5%) and performed comparably to GDs for periodontitis‐related tooth loss (76.2% vs. 76.3%). Calibration was substantially worse for AI (Brier score 0.247) than for periodontists and GDs (0.171, 0.170). Individually, AI performance overlapped with that of several clinicians.

**Conclusion:**

Clinicians outperformed AI under a fixed threshold, but AI performance was comparable to clinicians using optimal thresholds, situating AI within the range of experienced clinicians despite modest overall predictive ability.

## 1 Introduction

Establishing an accurate tooth prognosis is pivotal to effective treatment planning in patient‐centred Dentistry, particularly when long‐term stability of outcomes is a primary goal. Among the most complex decisions clinicians face is whether to retain or extract a periodontally compromised tooth (Kao 2008). While in some instances a clear clinical pathway is evident, many fall into a diagnostic ‘grey zone’ where making a decision is more challenging. In such scenarios, the ability to predict long‐term tooth survival is critical and may significantly influence treatment planning and patient quality of life.

Historically, tooth prognosis has relied on clinical experience and patient‐related local and systemic factors. Over the past five decades, several prognostic systems have been introduced to assist in clinical decision‐making (Becker et al. 1984; Hirschfeld and Wasserman 1978; Kwok and Caton 2007; McGuire and Nunn 1996; Miller et al. 2014). While these frameworks have advanced the field, they are not without limitations. For instance, some fail to account for systemic conditions like smoking and diabetes (Hirschfeld and Wasserman 1978; McGuire and Nunn 1996), which are now well‐established as critical modifiers of periodontal disease progression and treatment outcomes (Costa et al. 2013; Ravidà et al. 2020). Additionally, many frameworks rely heavily on subjective clinical judgement (Kwok and Caton 2007) and vary considerably in scope, with some focusing exclusively on specific tooth types, such as molars (Miller et al. 2014), limiting their generalizability in clinical practice. These challenges highlight the need for more objective and individualized approaches to establish individual tooth prognosis in modern periodontal care.

In recent years, the integration of artificial intelligence (AI) into healthcare has introduced new tools capable of integrating complex datasets and supporting clinical decision‐making. In dentistry, AI technologies have shown promising applications in endodontics, implant dentistry, oral radiology and increasingly, in the prediction of long‐term tooth survival (Oliveira et al. 2025; Mohammadnezhad et al. 2025; Kurt‐Bayrakdar et al. 2025; Khubrani et al. 2025; Fasih et al. 2025; Farina et al. 2025; Fanelli et al. 2025; Allihaibi et al. 2025; Troiano et al. 2024). AI‐based prognostic systems offer the potential to enhance consistency and reduce inter‐examiner variability, resulting in more objective decisions.

The first AI‐based model for predicting 10‐year tooth loss in periodontally affected patients was recently developed and published by our research group, trained and validated using clinical and radiographic data from four centres across three continents (two in Europe, one in the United States and one in China), including 3157 molars from 515 patients (Troiano et al. 2023). Among various approaches tested, the ensembled model combining a neural network and logistic regression performed best, with an area under the receiver operating characteristic (ROC) curve (AUC) of 0.726 and superior calibration. To extend generalizability beyond molars, the model was subsequently modified to include all tooth types and externally validated using data from two distinct cohorts (London and Pittsburgh) (Santamaria et al. 2024). The AI‐based model demonstrated high sensitivity (98.3%), moderate specificity (52.3%) and high positive predictive value (89.1%), confirming its potential for long‐term tooth loss prediction in clinical practice.

Despite rapid advancement and increasing clinical interest in AI‐based prognostic tools, it remains uncertain whether these models can match the predictive performance of experienced clinicians. While AI systems can integrate large volumes of structured clinical and radiographic data consistently, periodontal prognosis often relies on clinical judgement developed through years of experience. As AI technologies continue to evolve, direct comparisons with human expertise are essential to determine their true clinical value and limitations in real‐world decision‐making. Therefore, the objective of this study was to compare the predictive performance of an AI‐based model with that of a group of experienced clinicians in estimating tooth loss over a 10‐year period.

## 2 Materials and Methods

### 2.1 Study Design

This retrospective diagnostic accuracy study was conducted at the University of Pittsburgh School of Dental Medicine. Teeth with known 10‐year outcomes were identified from dental records, and clinical and radiographic information was extracted. Each selected tooth was independently evaluated by experienced clinicians using a standardized online form to estimate long‐term survival. These clinician‐derived prognostic assessments were compared with predictions generated by a previously developed AI‐based model applied to a predefined set of variables extracted from the same records. The protocol was approved by the University of Pittsburgh Institutional Review Board (protocol number 24030159). Given its retrospective nature and use of fully anonymized data, informed consent was not applicable. The manuscript was prepared in accordance with the STARD guidelines (Standards for Reporting of Diagnostic Accuracy Studies) (Cohen et al. 2016).

### 2.2 Participants

#### 2.2.1 Eligibility

Eligible patient records were identified through a retrospective database search.

Inclusion criteria were (1) at least one session of scaling and root planing performed in the department of Periodontics at the University of Pittsburgh School of Dental Medicine; (2) at least 10 years of follow‐up with a minimum of one periodontal maintenance visit per year; (3) baseline full‐mouth radiographs and a comprehensive periodontal charting; and (4) documented justification for any tooth extraction occurring during follow‐up.

Exclusion criteria included missing or inadequate baseline radiographs or periodontal charting, and cases where the reason for tooth extraction was unclear or unjustified. Extraction validity was confirmed independently by two investigators (G.A.‐O./A.R.), with disagreements resolved by consensus discussion.

#### 2.2.2 Data Mining

After applying the eligiblity criteria, a cohort of 117 patients (2841 teeth) was established. Patient‐ and tooth‐level data were extracted by two independent examiners (P.A.L.‐G./M.S.). Patient‐level variables included age, sex, smoking status (current/former/never; cigarettes/day) and diabetes diagnosis (yes/no). Tooth‐level variables included: clinical attachment loss, probing depth, tooth mobility, number of roots (single/multi‐rooted), furcation involvement, periapical index (Orstavik et al. 1986), previous endodontic treatment, presence of interproximal restorations, abutment tooth for a fixed dental prosthesis, percentage of radiographic bone loss (< 25%/25%–50%/> 50%), radiographic presence of vertical defects > 3 mm and tooth loss during the follow‐up period, including the reason for extraction. Data collection occurred from June 2024 to February 2025.

#### 2.2.3 Sample Categorization and Randomization Strategy

To ensure a balanced representation across different prognostic categories, all teeth were classified according to the Nibali classification system as ‘good’, ‘fair’, ‘questionable’, or ‘unfavourable’ (Nibali et al. 2017) (Figure 1). Due to the limited number of teeth classified as ‘unfavourable’, these were combined with ‘questionable’ into a single category. Additionally, all extracted teeth were categorized as lost due to periodontitis or due to other causes. Teeth were then selected using stratified randomization by prognostic category to yield a sample of 300 teeth, performed using an online tool (https://www.randomizer.org). A second stratified randomization step was applied to ensure representation of teeth lost due to periodontitis and due to other causes.

**FIGURE 1:**
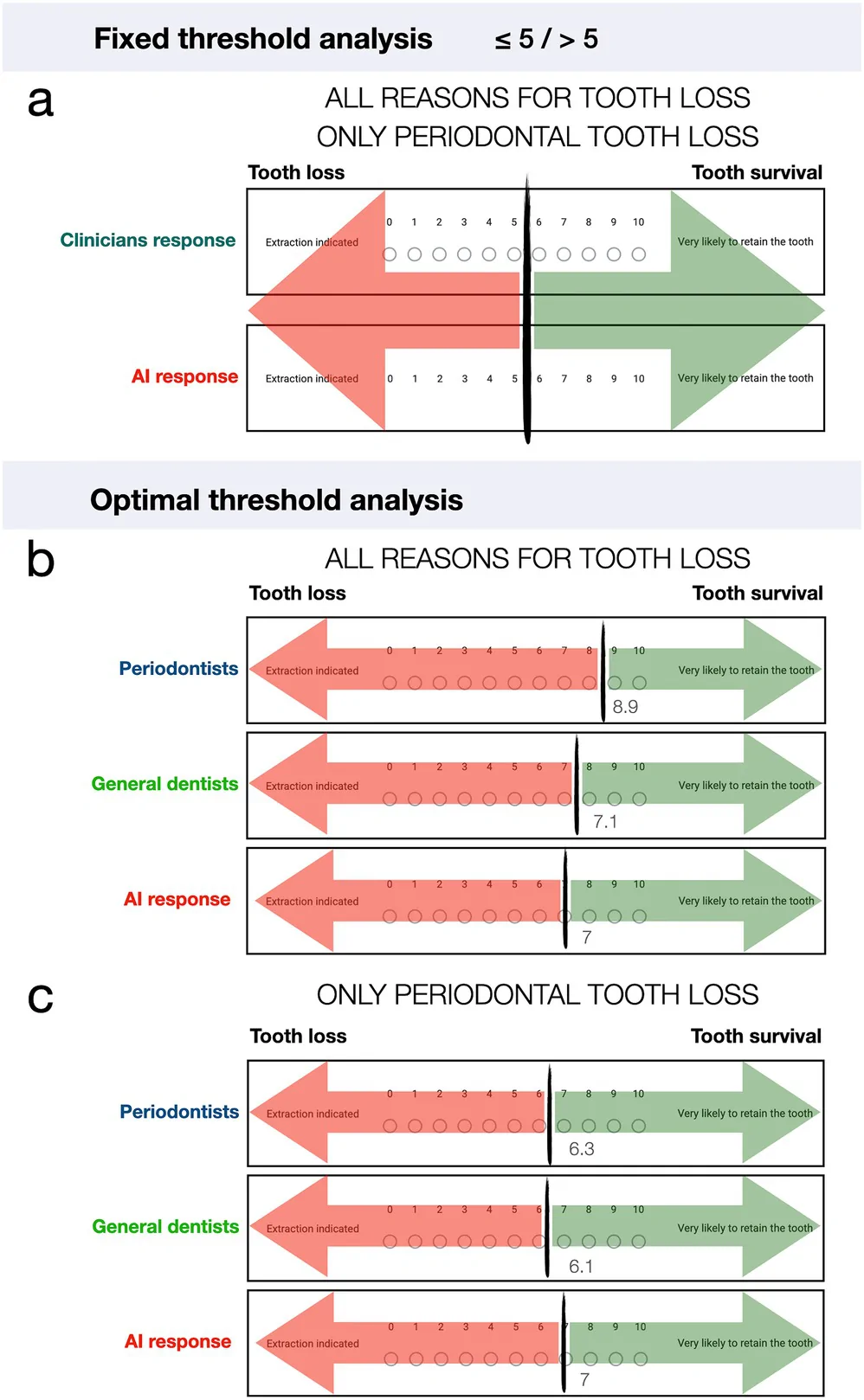
Thresholds utilized in the study. (a) Fixed threshold analysis using a cutoff of 5, applied to both all‐reason and periodontitis‐related tooth loss. (b,c) Optimized threshold analysis in which thresholds were determined separately for periodontists, GDs, and AI for teeth lost for any reason and teeth loss due to periodontitis.

### 2.3 Test Methods

#### 2.3.1 Clinicians' Evaluation

A total of 15 periodontists and 15 general dentists (GDs), each with ≥ 10 years of experience, were invited to serve as evaluators, with the aim of enrolling, at least, 10 clinicians per group. Clinicians were provided with general instructions, patient‐level data (age, smoking, diabetes), a panoramic or a set of full‐mouth periapical radiographs and a comprehensive periodontal chart. Then, they were asked to answer the question: ‘Do you think this tooth will survive for 10 years?’ using a scale from 0 to 10, where 0 indicated ‘immediate extraction indicated’ and 10 indicated ‘very likely to retain the tooth’. Full details of the clinician evaluation form, instructions, example scoring sheets and a summary of individual clinician responses are provided in the Supporting Information.

#### 2.3.2 AI‐Based Model

The AI model evaluated the same set of 300 teeth using structured clinical variables. Predictions were generated using a previously developed and validated neural network model (Santamaria et al. 2024). The model generated survival probabilities for each tooth, expressed as percentages ranging from 0% to 100%. These probabilities were then rounded to the nearest 10 and converted to a 0–10 ordinal scale to match the format used by the clinicians. A structured spreadsheet containing the variables was submitted to AI by a statistician (G.T.) who was blinded to the actual 10‐year outcomes.

### 2.4 Statistical Analyses

The primary objective of the present study was to compare the accuracy of tooth loss predictions among the AI model, periodontists and GDs, using observed 10‐year survival status as the reference standard. Sample size was calculated assuming moderate agreement (60% of cases both correct, 10% both incorrect) and 30% discordance (odds ratio 2 between discordant predictions; 20% vs. 10%). Using McNemar's test for paired binary outcomes (G*Power; 80% power, 95% confidence interval (CI); OR = 2, discordant pairs = 30%), a minimum of 250 teeth was required to detect a 10% difference in accuracy between two evaluators. To ensure sufficient power, 300 teeth were included.

Outcomes of interest included overall accuracy, sensitivity, specificity, positive predictive value (PPV), negative predictive value (NPV) and AUC. Secondary analyses evaluated inter‐rater reliability and calibration performance. Analyses were performed separately for tooth loss due to any cause and for tooth loss specifically due to periodontitis using complementary continuous‐ and threshold‐based approaches. Discriminative performance was first assessed using ROC curve analysis, which evaluated the ability of continuous 0–10 prognostic scores to distinguish between teeth that were lost and those that were retained, independent of any classification threshold. AUC point estimates and 95% CIs were calculated using DeLong's method, and AUC values were compared between evaluators using DeLong's test for paired ROC curves.

Subsequently, predicted scores were dichotomized into binary outcomes (tooth loss vs. survival) to evaluate clinically interpretable classification performance using two thresholding strategies:

- *Fixed threshold*: Scores from 0 to 10 were dichotomized using a predefined cut‐off, with values > 5 indicating predicted tooth survival, and values ≤ 5 indicating predicted tooth loss (Figure 1).
- *Optimized threshold*: Group‐specific optimal cut‐offs were calculated using Youden's Index to determine the optimal balance between sensitivity and specificity. For the AI model, the optimal decision threshold was adopted from the original development and validation study (AUC 0.7) (Santamaria et al. 2024). For the human control groups, optimized thresholds were derived using a 10‐fold cross‐validation framework, with each fold serving as a quasi‐validation set prior to performance evaluation (Figure 1).

Accuracy metrics were calculated for each clinician and group. Inter‐rater reliability within clinician groups was assessed using the intraclass correlation coefficient (ICC), applying a two‐way random‐effects model to account for evaluator‐ and tooth‐specific variability.

All statistical analyses were performed using R software (version 4.4.2) and SPSS (version 15.0). A significance level of *p* < 0.05 was adopted and 95% CIs were reported.

All statistical analyses were conducted by an independent expert statistician who was not involved in developing or applying the AI model.

## 3 Results

### 3.1 Participants

Of 330 screened patient records, 117 patients (2841 teeth) met eligibility criteria. After prognostic classification and stratified randomization, 300 teeth were selected; one was later excluded due to a slide error, yielding a final sample of 299 teeth from 45 patients (Figure 2).

**FIGURE 2:**
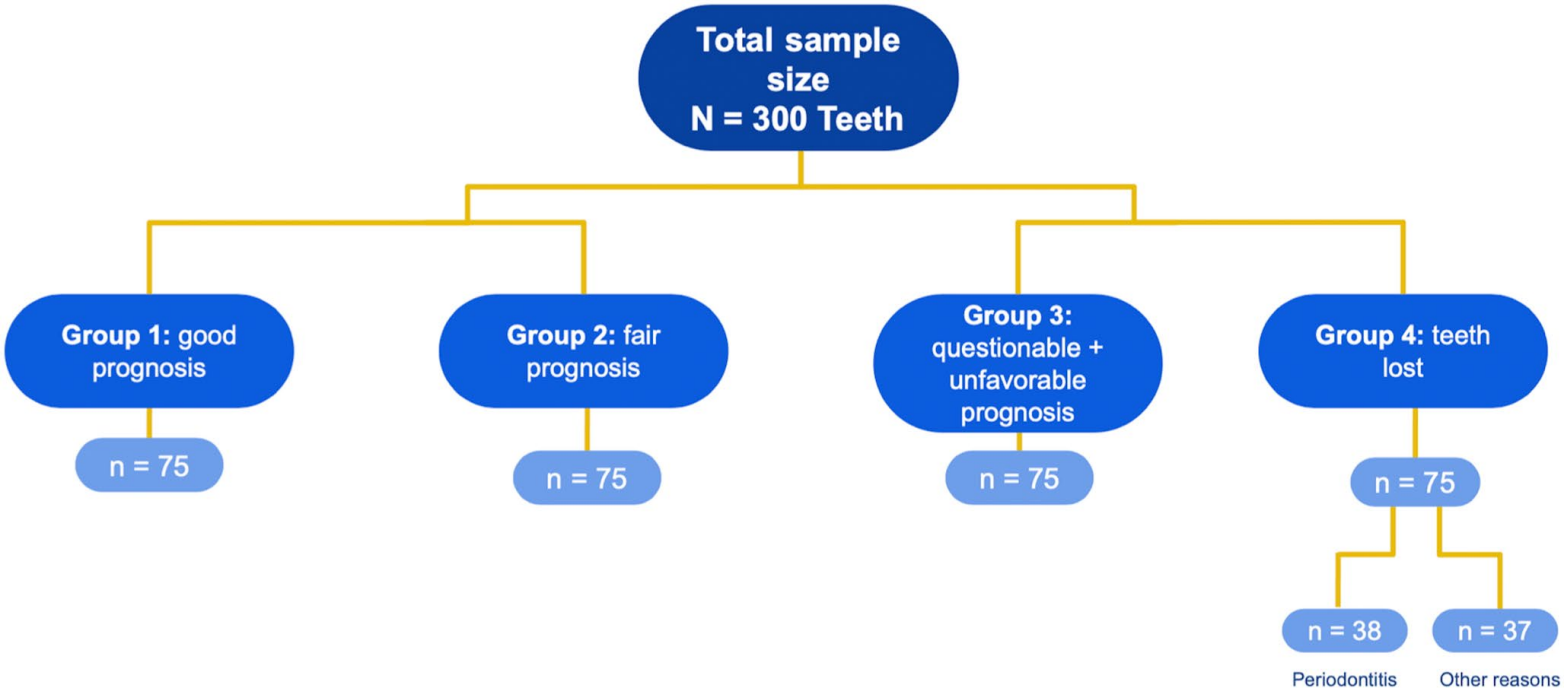
Flowchart displaying the distribution of teeth included in the study according to the classification proposed by Nibali et al. (2017).

### 3.2 Clinicians

A total of 12 periodontists and 11 GDs agreed to participate in the study. The inter‐rater reliability analysis showed moderate agreement among periodontists (ICC = 0.523, 95% CI: 0.432–0.604) and poor to moderate agreement among GDs (ICC = 0.460, 95% CI: 0.402–0.519).

### 3.3 ROC Curve Analysis

For tooth loss due to any cause, AUC was 0.714 (CI: 0.652–0.776) for periodontists, 0.711 (CI: 0.648–0.774) for GDs and 0.621 (CI: 0.550–0.693) for the AI model, with both clinician groups significantly outperforming AI (*p* < 0.01 and *p* < 0.05, respectively), and no difference between clinician groups (*p* > 0.05). When restricted to periodontitis‐related tooth loss, AUCs were higher and comparable across evaluators: 0.768 (CI: 0.687–0.849) for periodontists, 0.751 (0.665–0.837) for GDs and 0.751 (CI: 0.681–0.820) for AI, with no significant between‐group differences (all *p* > 0.05).

### 3.4 Analysis Using a Fixed Threshold

#### 3.4.1 Prediction of Tooth Loss for Any Cause (Periodontitis and Other Reasons)

Using a fixed threshold (> 5 = survival), mean accuracy was highest for periodontists (75.6%), followed by GDs (74.9%) and AI (69.2%), with both clinician groups outperforming AI (*p* = 0.015 and 0.022, respectively), and no difference between them (*p* > 0.05; Figure 3a). Sensitivity was low across all groups (14.7% periodontists, 22.7% GDs, 21.3% AI model; *p* > 0.05). Specificity was highest for periodontists (96%), then GDs (92.4%) and AI model (85.3%), with both clinician groups outperforming AI (*p* < 0.05; Figure 3a). PPV was 55% (periodontists), 50% (GDs) and 32.7% (AI; *p* > 0.05). NPV was similar across groups: 78.1% (GDs), 77.1% (periodontists) and 76.4% (AI; *p* > 0.05). Individually, three periodontists (Figure 3b) and one GD (Figure 3c) demonstrated higher accuracy than the AI model (*p* < 0.05), while the remaining clinicians did not differ significantly. All groups underestimated the true incidence of tooth loss (25%), though AI identified a greater proportion of lost teeth (16.3%) than GDs (11.2%) and periodontists (6.5%; Figure 3d).

**FIGURE 3:**
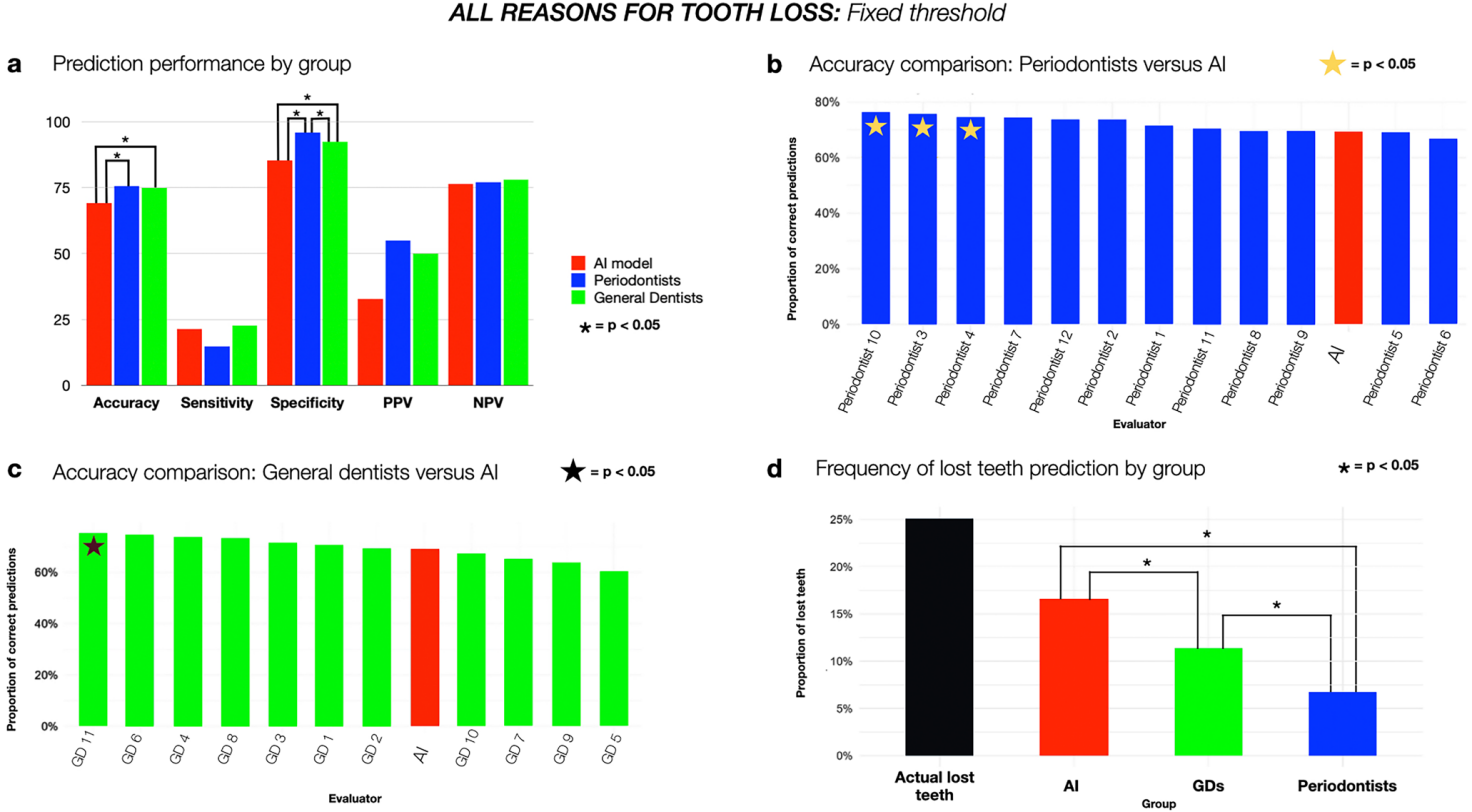
Overall accuracy analysis for all‐cause tooth loss, considering a score > 5 as the threshold to determine if the tooth survives. (a) Prediction performance by group. (b, c) Individual comparison between each of the enrolled periodontists (blue bars) or GDs (green bars) and the AI model (red bar). (d) Frequency of correct tooth loss prediction by group.

#### 3.4.2 Prediction of Tooth Loss due to Periodontitis

In the subgroup analysis limited to teeth lost due to periodontitis, mean accuracy was highest for periodontists (87.6%), followed by GDs (85.6%), and AI (79.3%), with both clinician groups outperforming AI (*p* < 0.05) and no difference between them (*p* = 0.264; Figure 4a). Sensitivity was highest for GDs (37.8%), then AI (32.4%) and periodontists (27%; *p* > 0.05). Specificity was highest for periodontists (96.2%), then GDs (92.4%) and AI (85.9%), with both clinician groups outperforming AI (*p* < 0.01) and periodontists outperforming GDs (*p* < 0.05; Figure 4a). PPV was higher for clinicians (50% periodontists, 41.2% GDs) than AI (24.4%; *p* > 0.05). NPV was high across groups, ranging from 90% (AI) to 91.3% (GDs) (*p* > 0.05; Figure 4a). Individually, six periodontists demonstrated significantly higher accuracy than AI, while one showed lower accuracy (*p* < 0.05; Figure 4b). Among GDs, one individual demonstrated higher accuracy, while three showed lower accuracy compared to the AI model (*p* < 0.05; Figure 4c). All evaluator groups underestimated the actual rate of tooth loss due to periodontitis. While 12.4% of teeth were ultimately lost for periodontal reasons, only 4.6% were predicted as such by GDs, 4% by the AI model and 3.6% by periodontists (Figure 4d).

**FIGURE 4:**
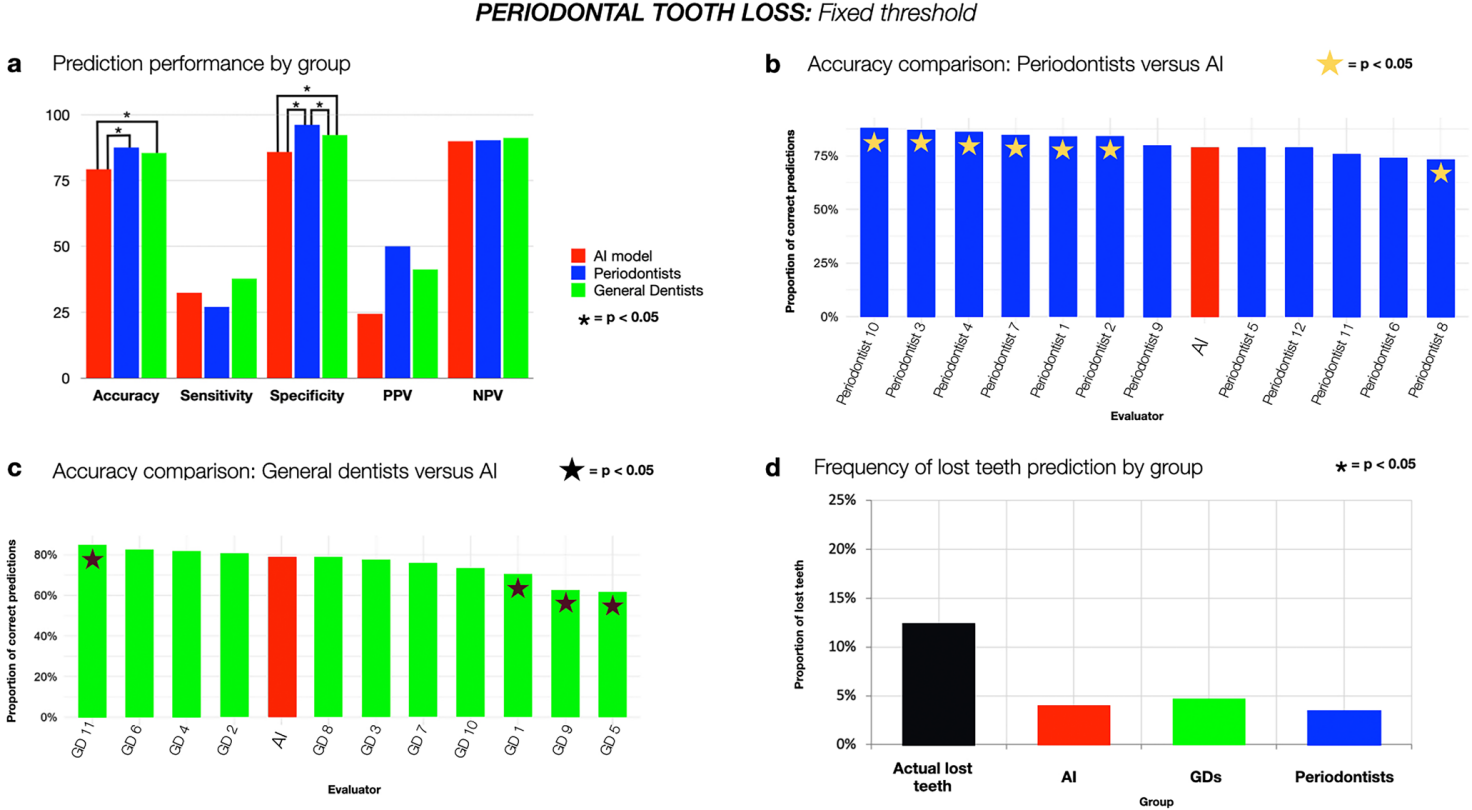
Overall accuracy analysis for teeth lost due to periodontitis with the threshold stablished at a score of 5. (a) Prediction performance by group. (b, c) Individual comparison between each of the enrolled periodontists (blue bars) or GDs (green bars) and the AI model (red bar). (d) Frequency of correct tooth loss prediction by group for teeth lost due to periodontitis.

### 3.5 Analysis Using Group‐Specific Optimal Thresholds

Optimal thresholds, derived using Youden's Index, differed by evaluator and outcome: 8.9 (periodontists), 7.1 (GDs) and 7 (AI) for all‐cause tooth loss; 6.3 (periodontists), 6.1 (GDs) and 7 (AI) for periodontitis‐related tooth loss.

#### 3.5.1 Prediction of Tooth Loss for Any Cause (Periodontitis and Other Reasons)

For all‐cause tooth loss, AI achieved the highest accuracy (67.5%), significantly outperforming periodontists (55.5%, *p* = 0.003) and GDs (57.2%, *p* = 0.004). Periodontists showed the highest sensitivity (94.7%) but lowest specificity (42.4%), while AI showed the inverse pattern (sensitivity 28%, specificity 80.8%; all *p* < 0.001). PPV was similar across groups (32.8%–35.5%); NPV was highest for periodontists (96%; Figure 5).

**FIGURE 5:**
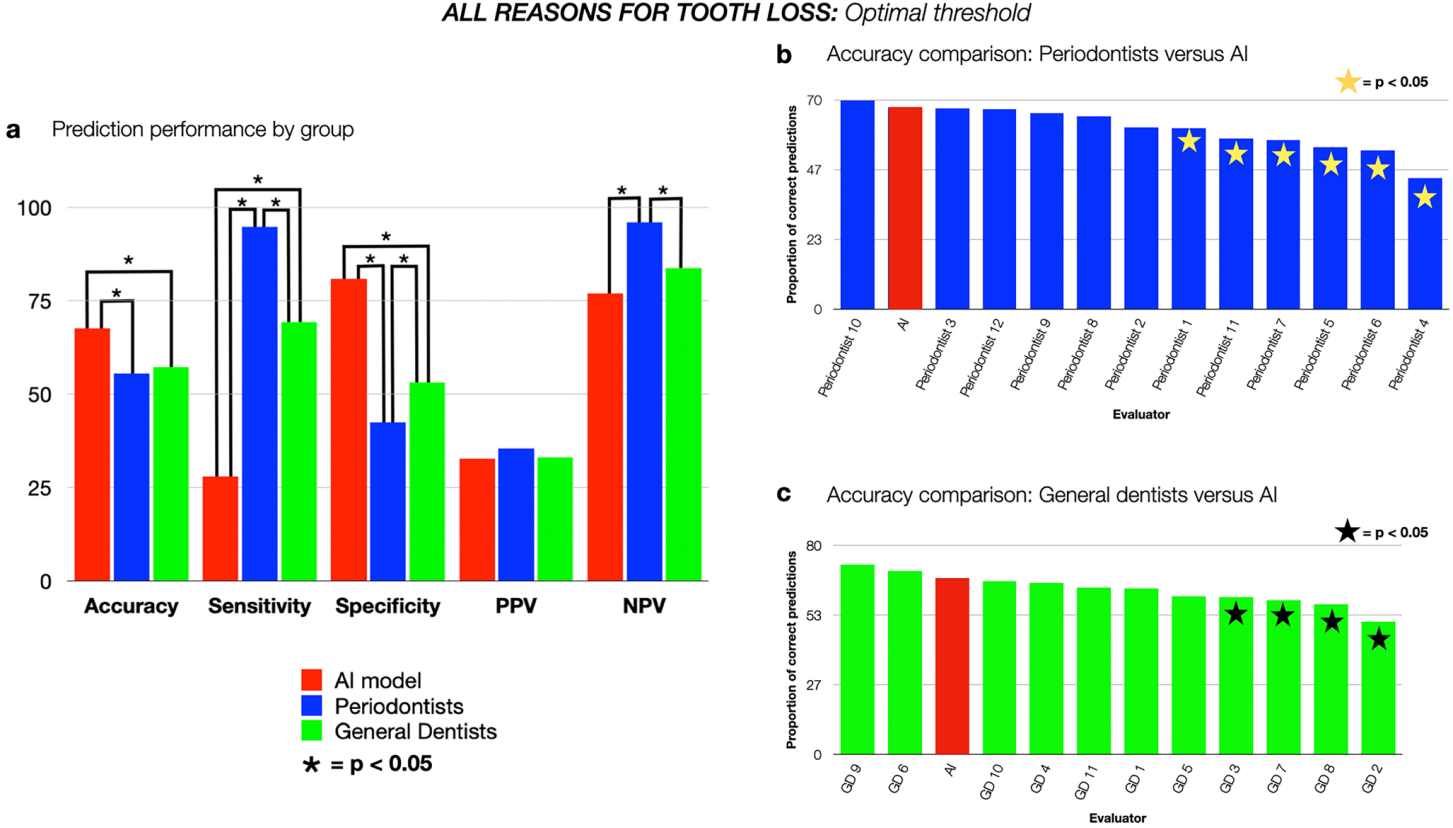
Overall accuracy analysis for all‐cause tooth loss, considering an optimal group‐specific threshold. (a) Prediction performance by group. (b, c) Individual comparison between each of the enrolled periodontists (blue bars) or GDs (green bars) and the AI model (red bar).

#### 3.5.2 Prediction of Tooth Loss due to Periodontitis

For periodontitis‐related tooth loss, AI (76.2%) and GDs (76.3%) achieved similar accuracy, both outperforming periodontists (66.9%, *p* < 0.001). Sensitivity did not differ significantly across groups, while specificity was lowest for periodontists (69.8%) and highest for AI (81.3%; Figure 6).

**FIGURE 6:**
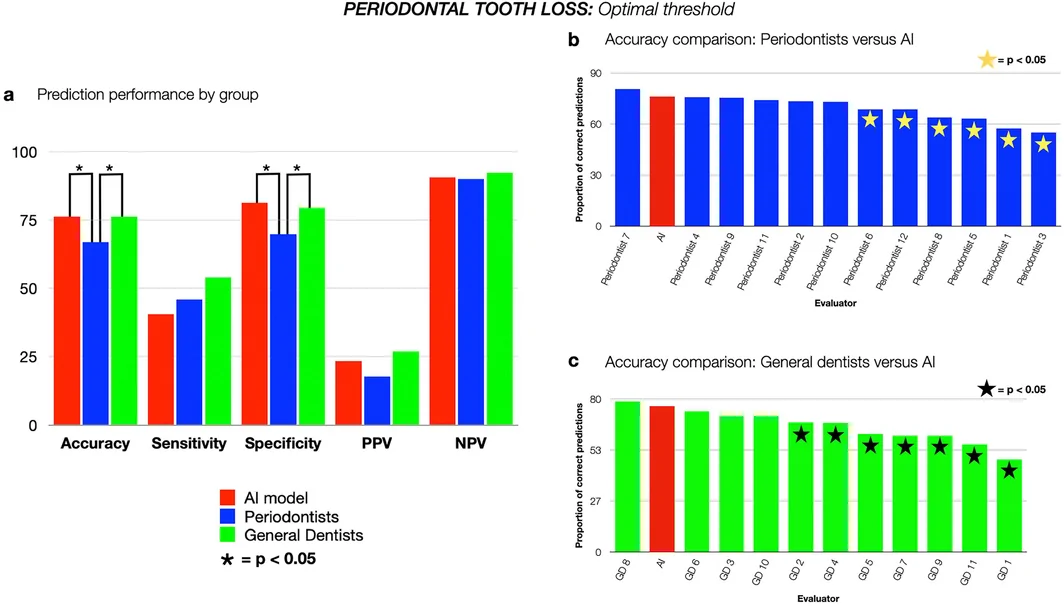
Overall accuracy analysis for teeth lost due to periodontitis with an optimal group‐specific threshold. (a) Prediction performance by group. (b, c) Individual comparison between each of the enrolled periodontists (blue bars) or GDs (green bars) and the AI model (red bar).

### 3.6 Calibration

Calibration was poorest for the AI model, indicating substantial miscalibration and overestimation of tooth survival probabilities (Figure 7). Periodontists showed smaller deviations from ideal calibration, whereas GDs demonstrated the closest agreement between predicted and observed outcomes.

**FIGURE 7:**
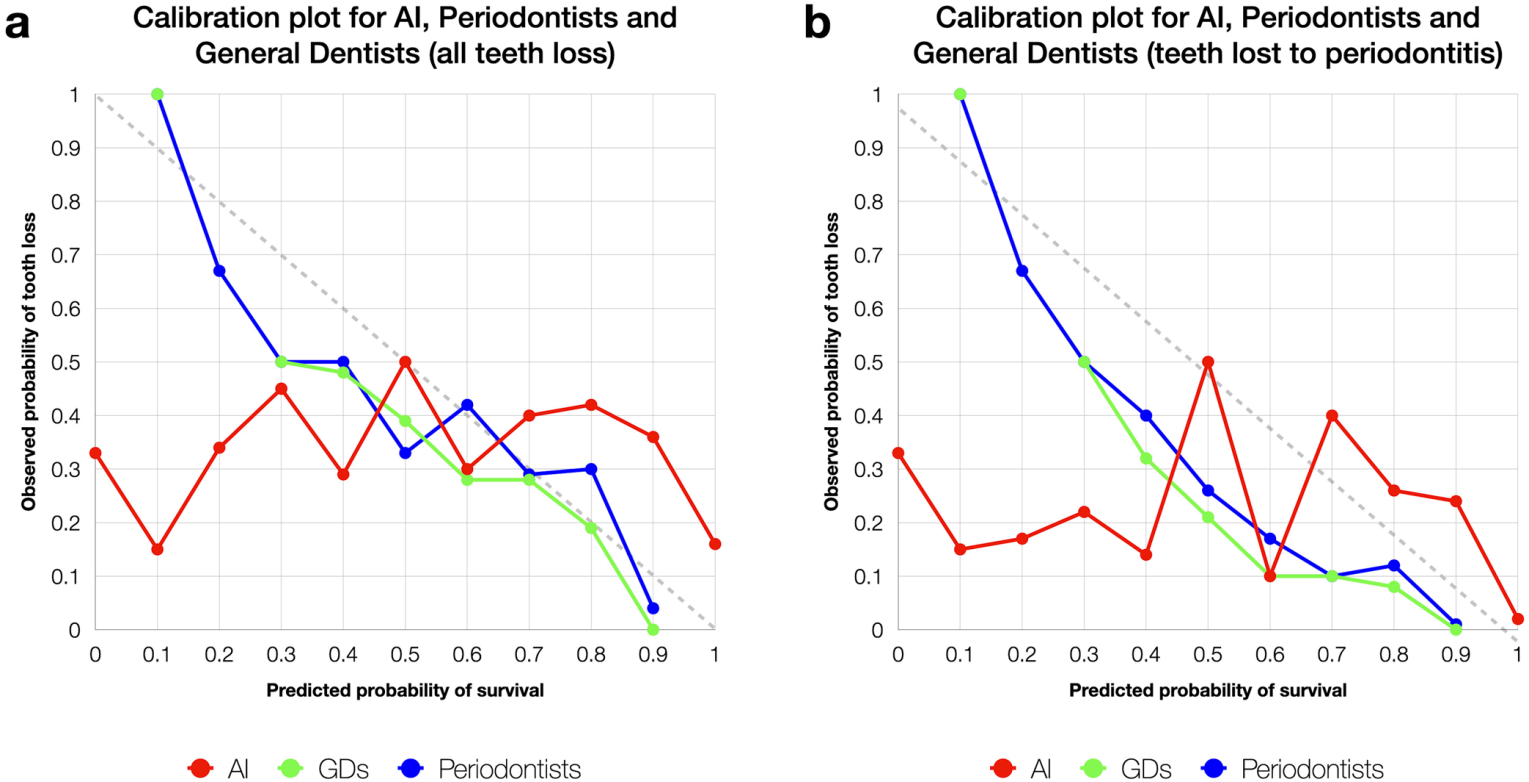
Calibration plot to assess the agreement between predicted probabilities and observed outcomes for both tooth loss due to any cause. (a) Teeth loss by any reasons: AI tended to overestimate survival, particularly at the lower and higher ends of the probability range, leading to an underestimation of actual tooth loss. Periodontists consistently overestimated the risk of tooth loss in cases with low predicted survival. GDs showed better calibration overall, especially in the mid‐probability range, although some deviation remained at the extremes. (b) Teeth loss due to periodontal disease: Calibration improved across all groups. The AI model showed more accurate predictions in the mid‐range, while GDs demonstrated the most stable and consistent calibration throughout the probability spectrum. Periodontists still slightly overestimated risk at lower predicted survival probabilities, but their calibration also improved compared to the analysis of tooth loss due to any cause. The dashed line represents perfect calibration, where predicted probabilities align exactly with observed outcomes. Since the model predicts the probability of tooth survival rather than loss, the reference line is inverted, reflecting that when the probability of survival is 0, the probability of loss is 1.

Additional statistical details as well as supporting figures and tables are provided in the Supporting Information.

## 4 Discussion

This study is the first to compare the performance of an AI model with that of experienced clinicians in predicting 10‐year tooth loss from clinical and radiographic parameters. Given this novel design, direct comparisons with earlier studies are limited. Prognostic performance was evaluated using complementary continuous and threshold‐based approaches, stratified by all‐cause and periodontitis‐related tooth loss. ROC analyses based on continuous prognostic scores showed higher discrimination for clinicians in predicting all‐cause tooth loss, whereas discrimination improved and became comparable across clinicians and AI for periodontitis‐related outcomes.

Using a fixed threshold (> 5 = survival), periodontists achieved the highest accuracy and specificity, followed by GDs, both outperforming AI for all‐cause and periodontitis‐related tooth loss. Sensitivity remained low across all groups, indicating systematic underestimation of the true tooth loss incidence, with AI more frequently identifying teeth that were actually lost. These findings are consistent with prior literature evaluating clinician‐based prognostic systems for periodontitis‐related tooth loss, where sensitivity has been reported to be as low as 3%–13% despite high specificity (Saydzai et al. 2022). This fixed‐threshold approach, based on a midpoint cut‐off on a 0–10 scale, mirrors common clinical reasoning but compresses graded risk into binary decisions: borderline scores (e.g., 6) are classified as ‘survival’ despite acknowledged risk, and subsequent loss is counted as misclassification, contributing to the modest sensitivities observed.

When evaluator‐specific optimal thresholds were applied, performance patterns shifted by outcome. For all‐cause tooth loss, AI achieved the highest accuracy, driven by substantially higher specificity, whereas periodontists maintained the highest sensitivity at the expense of specificity. AI's higher accuracy was accompanied by markedly low sensitivity (28% for all‐cause tooth loss), meaning it failed to identify most teeth ultimately lost, though clinically this trade‐off limits harm from over‐treatment (driven by false positives) more than it creates direct risk from missed cases. For periodontitis‐related tooth loss, accuracy was comparable between AI and GDs and higher than that of periodontists, while sensitivity differences across groups were attenuated. These findings indicate optimized thresholds primarily reflect evaluator‐specific decision strategies rather than intrinsic differences in prognostic ability. Given the moderate inter‐rater agreement among clinicians, fixed midpoint cut‐offs likely magnify differences between conservative and less conservative scorers, whereas optimized thresholds recalibrate decision boundaries to each evaluator's own scoring tendencies (e.g., if clinicians reserve ‘loss’ for scores ≤ 2, using that cut‐off better reflects their reasoning and improves sensitivity). The differing results across thresholding strategies should therefore be seen not as contradictory, but reflecting the trade‐off between real‐world applicability and theoretical benchmarking.

Across all analyses, tooth loss attributable to periodontitis was more accurately classified than tooth loss due to heterogeneous causes. Under the fixed‐threshold approach, mean accuracy increased from approximately 70% to 76% for all‐cause tooth loss to 80%–87% for periodontitis‐related tooth loss across evaluators. A similar pattern was observed under optimized thresholds, with accuracy improving from 55% to 68% for all‐cause tooth loss to 67%–76% for periodontitis‐related outcomes. These results suggest tooth loss driven by periodontitis may be more consistently classified than tooth loss due to heterogeneous causes.

When individual clinicians were examined, the AI performance fell within the same range as most clinicians across both thresholding approaches. Under the fixed threshold, only a small subset of clinicians outperformed the AI in terms of accuracy (three periodontists and one GD for all‐cause tooth loss; six periodontists and one GD for periodontitis‐related tooth loss), with the majority performing comparably. Under optimized thresholds, inter‐clinician differences became more pronounced, and for periodontitis‐related tooth loss in particular, AI performed as well as or better than most individual clinicians, with none outperforming AI across both outcomes. Overall, AI performance was comparable to that of individual clinicians rather than consistently superior or inferior, underscoring substantial variability in prognostic accuracy among clinicians.

Calibration, the agreement between predicted probabilities and observed outcomes, is a key but often overlooked aspect of model performance: poor calibration means that even models with high AUCs may systematically over‐ or underestimate risk, reducing their clinical reliability. In the present study, all groups, including AI, tended to underestimate the true rate of tooth loss, particularly at the extremes of the prediction spectrum, with AI showing worse calibration than the clinicians. This underscores the need for periodic recalibration of AI tools as they are applied to new populations and clinical contexts.

This study has several limitations. Although the AI model was previously developed and externally validated per TRIPOD guidelines (Santamaria et al. 2024), its performance here may have been influenced by variations in treatment philosophy, documentation quality and population characteristics across multiple treating clinicians, introducing heterogeneity despite enhancing generalizability. A single‐clinician, standardized cohort would likely improve internal consistency at the expense of generalizability. Data availability also differed between evaluators: AI relied on a limited structured variables, whereas clinicians accessed comprehensive periodontal charts and full‐mouth radiographs, though neither had extra‐ or intraoral photographs, models, occlusal information or face‐to‐face interactions, and the AI model lacked raw radiographic imaging or radiomic features that might capture subtle anatomical/pathological cues. Incorporating full imaging datasets into future AI systems could improve diagnostic granularity. Finally, the participating clinicians were highly experienced, likely representing the upper range of prognostic performance; AI tools may play a more impactful role in settings with greater variability in clinician training.

This study did not aim to equate AI with the full depth of clinician judgement, but to explore how far a model trained on structured variables alone can provide reliable prognostic insight. Even with limited inputs, AI could identify teeth at elevated risk, particularly in borderline cases, complementing rather than replacing human expertise. Because prognostic decisions are often subjective and shaped by factors beyond biological parameters, AI is best regarded as a decision‐support tool, enhancing accuracy and reducing time and data demands, improving consistency in treatment planning and facilitating risk communication with patients, while clinicians retain responsibility for contextualizing predictions within the broader clinical picture. Future studies should incorporate multimodal inputs and improve calibration so predicted risks more closely reflect real‐world outcomes (Santamaria et al. 2024; Saydzai et al. 2022).

## 5 Conclusion

When a fixed threshold was applied, clinicians demonstrated higher overall accuracy in predicting tooth loss over a 10‐year period. At the individual level, for all‐cause tooth loss, a limited number of clinicians (3/12 periodontists and 1/11 GDs) achieved higher accuracy than the AI model, whereas 19 clinicians showed no statistically significant differences compared with AI. Focusing only on tooth loss due to periodontitis, 6/12 periodontists and 1 GD outperformed the model, while 1 periodontist and 3 GDs were outperformed by AI; the remaining clinicians did not differ significantly from AI. Although long‐term tooth prognosis is difficult, AI performed within the range of clinicians despite modest predictive ability. AI may serve as a supportive decision‐support tool by assisting in the identification of high‐risk teeth and promoting data‐driven treatment planning, particularly in borderline cases or among less experienced clinicians.

## Author Contributions

Pablo Antonio López‐Galindo conceived and designed the study, collected data, contributed to data analysis and interpretation and drafted the manuscript. Giuseppe Troiano contributed to data analysis and interpretation and critically reviewed the manuscript. Matteo Serroni participated in data collection and drafting of the manuscript. Debora R. Dias contributed to data analysis and interpretation and drafting of the manuscript. Pasquale Santamaría and Luigi Nibali critically reviewed the manuscript. Gustavo Avila‐Ortiz was involved in study conception and design, data interpretation and critically reviewed the manuscript. Andrea Ravidà conceived and designed the study, contributed to data analysis and interpretation and participated in drafting the manuscript.

## Funding

The authors have nothing to report.

## Conflicts of Interest

The authors declare no conflicts of interest.

## Supporting information


**Data S1:** Material and methods and results.
**Table S1:** Evaluators demographic data.
**Figure S1:** Representative example of the form sent to the clinicians.
**Table S2:** Patient‐level demographic data.
**Table S3:** Tooth‐level clinical data.
**Figure S2:** Distribution of tooth loss causes among those that were lost.
**Table S4:** Periodontists: Score for survival (0–10).
**Table S5:** General dentists: Score for survival (0–10).
**Table S6:** AI: Score for survival (0–10).
**Table S7:** ROC analysis for AI, periodontists and general dentists.
**Table S8:** Pairwise DeLong test for AUC comparison between AI, periodontists and general dentists predicting tooth loss due to any causes or due to periodontitis.
**Table S9:** Periodontists: Predicted tooth survival versus loss (teeth with score > 5 were considered predictions as ‘saved’; teeth with score ≤ 5 were considered predictions as ‘lost’).
**Table S10:** General dentists: Predicted tooth survival versus loss (teeth with score > 5 were considered predictions as ‘saved’; teeth with score ≤ 5 were considered predictions as ‘lost’).
**Table S11:** AI: Predicted tooth survival versus loss (teeth with score > 5 were considered predictions as ‘saved’; teeth with score ≤ 5 were considered predictions as ‘lost’).
**Table S12:** Comparison of accuracy, sensitivity and specificity between AI, periodontists and general dentists: McNemar's test *χ* ^2^, degrees of freedom and *p*‐values for pairwise comparisons.
**Table S13:** Comparison of accuracy between AI and individual periodontists: McNemar's test *χ* ^2^, degrees of freedom and *p*‐values.
**Table S14:** Comparison of accuracy between AI and individual general dentists: McNemar's test *χ* ^2^, degrees of freedom and *p*‐values.
**Table S15:** Comparison of accuracy, sensitivity and specificity between AI, periodontists and general dentists: McNemar's test *χ* ^2^, degrees of freedom and *p*‐values for pairwise comparisons.
**Table S16:** Comparison of accuracy between AI and individual periodontists: McNemar's test *χ* ^2^, degrees of freedom and *p*‐values.
**Table S17:** Comparison of accuracy between AI and individual general dentists: McNemar's test *χ* ^2^, degrees of freedom and *p*‐values.
**Table S18:** Periodontists: Predicted tooth survival versus loss (teeth with score > 8.9 were considered predictions as ‘saved’; teeth with score ≤ 8.9 were considered predictions as ‘lost’).
**Table S19:** General dentists: Predicted tooth survival versus loss (teeth with score > 7.1 were considered predictions as ‘saved’; teeth with score ≤ 7.1 were considered predictions as ‘lost’).
**Table S20:** AI: Predicted tooth survival versus loss (teeth with score > 7 were considered predictions as ‘saved’; teeth with score ≤ 7 were considered predictions as ‘lost’).
**Table S21:** Comparison of accuracy, sensitivity and specificity between AI, periodontists and general dentists: *p*‐values for pairwise comparisons using McNemar's test.
**Table S22:** Comparison of accuracy between AI and individual periodontists: *p*‐values for McNemar's test.
**Table S23:** Comparison of accuracy between AI and individual general dentists: *p*‐values for McNemar's test.
**Table S24:** Comparison of accuracy, sensitivity and specificity between AI, periodontists and general dentists: *p*‐values for pairwise comparisons using McNemar's test.
**Table S25:** Comparison of accuracy between AI and individual periodontists: *p*‐values for McNemar's test.
**Table S26:** Comparison of accuracy between AI and individual general dentists: *p*‐values using McNemar's test.
**Table S27:** Calibration performance of the AI model and human raters for 10‐year tooth loss prediction.

## Data Availability

Data are available upon request to the corresponding author.
