# Supplementary material for: Can Artificial Intelligence Match Human Expertise in Long‐Term Periodontal Prognosis? A Comparative Accuracy Study
Source: J Clin Periodontol. 2026 Aug 21;53(11):1712–21. doi: 10.1111/jcpe.70191 (PMC13658045; doi:10.1111/jcpe.70191)
Supplement: Supplementary file 1 [file JCPE-53-1712-s001.docx]

**Can Artificial Intelligence Match Human Expertise in Long-Term Periodontal Prognosis? A Comparative Accuracy Study**

Pablo Antonio López-Galindo, Giuseppe Troiano, Matteo Serroni, Debora R. Dias, Pasquale Santamaría, Luigi Nibali, Gustavo Avila-Ortiz & Andrea Ravidà

**SUPPLEMENTARY MATERIAL**

[2. MATERIAL AND METHODS	3](#_Toc235901839)

[2.3. Test methods	3](#_Toc235901840)

[2.3.1. Clinicians’ evaluation	3](#_Toc235901841)

[Supplementary Table 1. Evaluators Demographic Data.	3](#_Toc235901842)

[Supplementary Figure 1. Representative example of the form sent to the clinicians.	5](#_Toc235901843)

[2.3.2. AI-based model	6](#_Toc235901844)

[Neural Network-Based Prognostic Model	6](#_Toc235901845)

[2.4. Statistical analysis	7](#_Toc235901846)

[2.4.1. Calibration	7](#_Toc235901847)

[3. RESULTS	7](#_Toc235901848)

[3.1. Participants	7](#_Toc235901849)

[Supplementary Table 2. Patient-Level Demographic Data.	8](#_Toc235901850)

[Supplementary Table 3. Tooth-Level Clinical Data.	8](#_Toc235901851)

[Supplementary Figure 2. Distribution of tooth loss causes among those that were lost.	9](#_Toc235901852)

[3.2. Clinicians’ evaluation and Artificial intelligence	10](#_Toc235901853)

[Supplementary Table 4. Periodontists: Score for Survival (0–10).	10](#_Toc235901854)

[Supplementary Table 5. General Dentists: Score for Survival (0–10).	10](#_Toc235901855)

[Supplementary Table 6. AI: Score for Survival (0–10).	11](#_Toc235901856)

[3.3. ROC curve analysis	12](#_Toc235901857)

[Supplementary Figure 3. ROC curve analysis comparing the diagnostic performance of periodontists, GDs, and the AI model.	12](#_Toc235901858)

[Supplementary Table 7. ROC Analysis for AI, periodontists and general dentists.	13](#_Toc235901859)

[Supplementary Table 8. Pairwise DeLong Test for AUC Comparison between AI, periodontists, and general dentists predicting tooth loss due to any causes or due to periodontitis.	13](#_Toc235901860)

[3.4. Analysis using a fixed threshold	13](#_Toc235901861)

[Supplementary Table 9. Periodontists: Predicted Tooth Survival vs. Loss (Teeth with score > 5 were considered predictions as ‘saved’; Teeth with score ≤ 5 were considered predictions as ‘lost’).	13](#_Toc235901862)

[Supplementary Table 10. General Dentists: Predicted Tooth Survival vs. Loss (Teeth with score > 5 were considered predictions as ‘saved’; Teeth with score ≤ 5 were considered predictions as ‘lost’).	14](#_Toc235901863)

[Supplementary Table 11. AI: Predicted Tooth Survival vs. Loss (Teeth with score > 5 were considered predictions as ‘saved’; Teeth with score ≤ 5 were considered predictions as ‘lost’).	15](#_Toc235901864)

[3.4.1. Prediction of tooth loss for any cause (periodontitis and other reasons)	15](#_Toc235901865)

[Supplementary Table 12. Comparison of accuracy, sensitivity and specificity between AI, periodontists, and general dentists.	15](#_Toc235901866)

[Supplementary Table 13. Comparison of accuracy between AI and individual periodontists	15](#_Toc235901867)

[Supplementary Table 14. Comparison of accuracy between AI and individual general dentists	16](#_Toc235901868)

[3.4.2. Prediction of tooth loss due to periodontitis	16](#_Toc235901869)

[Supplementary Table 15. Comparison of accuracy, sensitivity and specificity between AI, periodontists, and general dentists	17](#_Toc235901870)

[Supplementary Table 16. Comparison of accuracy between AI and individual periodontists	17](#_Toc235901871)

[Supplementary Table 17. Comparison of accuracy between AI and individual general dentists	17](#_Toc235901872)

[3.5. Analysis using group-specific optimal thresholds	18](#_Toc235901873)

[Supplementary Table 18. Periodontists: Predicted Tooth Survival vs. Loss (Teeth with score > 8.9 were considered predictions as ‘saved’; Teeth with score ≤ 8.9 were considered predictions as ‘lost’).	19](#_Toc235901874)

[Supplementary Table 19. General Dentists: Predicted Tooth Survival vs. Loss (Teeth with score > 7.1 were considered predictions as ‘saved’; Teeth with score ≤ 7.1 were considered predictions as ‘lost’).	19](#_Toc235901875)

[Supplementary Table 20. AI: Predicted Tooth Survival vs. Loss (Teeth with score > 7 were considered predictions as ‘saved’; Teeth with score ≤ 7 were considered predictions as ‘lost’).	20](#_Toc235901876)

[Supplementary Table 21. Comparison of accuracy, sensitivity and specificity between AI, periodontists, and general dentists	20](#_Toc235901877)

[Supplementary Table 22. Comparison of accuracy between AI and individual periodontists	20](#_Toc235901878)

[Supplementary Table 23. Comparison of accuracy between AI and individual general dentists	21](#_Toc235901879)

[Supplementary Table 24. Comparison of accuracy, sensitivity and specificity between AI, periodontists, and general dentists	22](#_Toc235901880)

[Supplementary Table 25. Comparison of accuracy between AI and individual periodontists.	22](#_Toc235901881)

[Supplementary Table 26. Comparison of accuracy between AI and individual general dentists	23](#_Toc235901882)

[3.6. Calibration performance	23](#_Toc235901883)

[Supplementary Table 27.	23](#_Toc235901884)

# 2. MATERIAL AND METHODS

## 2.3. Test methods

### 2.3.1. Clinicians’ evaluation

Fifteen periodontists and fifteen general dentists (GD), each with ≥ 10 years of experience, were invited to participate, with the aim of enrolling 10 clinicians in each group. Invitations were sent via email, and those who agreed to participate received a follow-up message with individualized access to the evaluation materials. To ensure anonymity and prevent bias, evaluator identities were numerically coded, and their responses were randomized before data analysis.

Of the 12 periodontists who completed the evaluation, all hold academic appointments or work full-time in academia, and 7 also have experience in private practice (Supplementary Table 1). Among the 11 general dentists who participated as assessors, 6 have academic appointments and 10 have experience in private practice (Supplementary Table 1).

#### Supplementary Table 1. **Evaluators Demographic Data.**

| **Periodontist Evaluator nº** | **Academy** | **Private Practice** |
| --- | --- | --- |
| 1 | Yes | No |
| 2 | Yes | Yes |
| 3 | Yes | No |
| 4 | Yes | Yes |
| 5 | Yes | No |
| 6 | Yes | No |
| 7 | Yes | Yes |
| 8 | Yes | Yes |
| 9 | Yes | Yes |
| 10 | Yes | Yes |
| 11 | Yes | Yes |
| 12 | Yes | Yes |
| **General Dentist Evaluator Nº** | **Academy** | **Private Practice** |
| 13 | No | Yes |
| 14 | Yes | Yes |
| 15 | Yes | Yes |
| 16 | Yes | Yes |
| 17 | No | Yes |
| 18 | Yes | No |
| 19 | Yes | Yes |
| 20 | No | Yes |
| 21 | Yes | Yes |
| 22 | No | Yes |
| 23 | No | Yes |

Clinicians were asked to respond to the question: “Do you think this tooth will survive for 10 years?” using a scale from 0 to 10, where 0 indicated “immediate extraction indicated” and 10 indicated “very likely to retain the tooth”. A representative example of the form is shown in the Supplementary Figure 1. Periodontal charts included site-specific information such as probing pocket depth, position of the mucosal margin in relation to the cementoenamel junction, clinical attachment loss, bleeding on probing, mobility, furcation involvement, keratinized tissue width, and presence of plaque. Radiographs were provided to evaluate periapical pathology, endodontic treatment status, presence of interproximal restorations, function as an abutment tooth, presence of vertical bone defects, and estimated percentage of bone loss. To reflect clinical reality, no specific criteria were imposed on evaluators regarding which parameters to prioritize in their decision-making.

Clinicians were asked to complete their evaluations within one month. Upon completion, one researcher (P.L.G.) cross-referenced the email addresses used to access the forms with the evaluator list to verify identity.

#### Supplementary Figure 1. Representative example of the form sent to the clinicians.

### 2.3.2. AI-based model

To evaluate the same set of teeth, a structured spreadsheet was compiled and submitted to a statistician (G.T.), who was blinded to the actual tooth outcomes. Included variables were patient age (at baseline), gender, smoking status (current/former/never), periapical index, endodontic treatment status, presence of interproximal restorations, function as an abutment tooth, furcation involvement, probing pocket depth, clinical attachment loss, tooth mobility, presence of vertical defects deeper than 3 mm, and radiographic bone loss classification. The author (G.T.) used the structured dataset as input to obtain predictions from a previously developed and validated AI-based neural network model (Santamaria et al., 2024). The model independently generated survival probabilities for each tooth, expressed as percentages ranging from 0% to 100%. These probabilities were then rounded to the nearest ten and converted to a 0–10 ordinal scale to match the format used by the clinicians.

##### Neural Network-Based Prognostic Model

To develop the prediction model, a dataset from University of Pittsburgh containing key clinical parameters was used. The involved clinical features were furcation involvement, probing pocket depth (PPD), tooth mobility, periapical status and percentage of bone loss, with 10-year tooth loss (10y TL) as the dependent outcome. After data cleaning, categorical variables were standardized and encoded to ensure proper handling by the algorithm. We then constructed a multilayer perceptron (MLP) neural network classifier, optimizing its structure and parameters through grid search with tenfold cross-validation. The hyperparameter search explored different activation functions, solvers, hidden layer architectures, and regularization strengths, ultimately identifying the best configuration as a two-hidden-layer network with 50 neurons each, rectified linear unit (ReLU) activation, Adam optimizer, and alpha = 0.01.

The training dataset was randomly split into a development and validation subset, on which the tuned model was internally evaluated. Discriminative ability was quantified by the area under the receiver operating characteristic curve (AUC), while accuracy, sensitivity, and specificity were also calculated. In this phase, the model showed excellent performance, with an AUC close to 0.90 and overall accuracy above 0.90, though sensitivity remained relatively low compared to specificity. Model calibration was further investigated by plotting predicted probabilities against observed outcomes, and threshold analyses were performed to explore the balance between sensitivity and specificity at different cut-off values.

For external validation, the model was applied to an independent dataset from the King’s College of London prepared using the same preprocessing strategy. Importantly, specificity remained high (>0.96), while sensitivity improved compared to the development phase. Calibration and sensitivity/specificity curves were again plotted to ensure the stability of the model across populations. All steps, from preprocessing to model training and validation, were implemented in Python (scikit-learn framework).

## 2.4. Statistical analysis

### 2.4.1. Calibration

Calibration was assessed by grouping predicted probabilities into deciles and comparing them to observed tooth loss rates. Calibration curves were generated for AI, periodontists, and general dentists. The ideal calibration line (a 45-degree diagonal) represented perfect agreement between predicted and observed outcomes; deviations from this line indicated miscalibration. Group-level comparisons between clinicians and the AI model were the primary focus of analysis.

# 3. RESULTS

## 3.1. Participants

A total of 330 patient electronic records were initially screened. Of these, 213 were excluded: 102 due to missing baseline full-mouth radiographs or incomplete periodontal charts, 71 due to lack of compliance with supportive periodontal care, and 40 due to insufficient follow-up. A total of 117 patients met all eligibility criteria, contributing with 2959 teeth for initial assessment (average of 23 teeth per patient). After prognostic evaluation using the Nibali classification system, 1008 teeth were classified as "good," 751 as "fair," 532 as "questionable," and 15 as "unfavorable." Additionally, 511 teeth were identified as lost during the follow-up period, of which 139 were extracted due to periodontitis and 372 due to other causes. Following stratified randomization, 300 teeth were selected for comparative analysis (Figure 2). After completion of the assessment by the clinicians, one tooth was excluded from the final analysis due to an error in the evaluation slide description, resulting in a final sample of 299 teeth from 45 patients. Accordingly, the final distribution was as follows: Group 1 (good prognosis), 75 teeth; Group 2 (fair prognosis), 75 teeth; Group 3 (questionable + unfavorable prognosis), 74 teeth; and Group 4 (lost teeth), 75 teeth. Among the teeth lost during follow-up, 23 had a good prognosis, 18 a fair prognosis, 33 a questionable prognosis, and 1 an unfavorable prognosis at baseline.

Baseline characteristics are presented in Supplementary Table 2 and 3. Reasons for tooth loss are shown in Supplementary Figure 2.

#### Supplementary Table 2. **Patient-Level Demographic Data.**

|  | **Overall** |
| --- | --- |
| **Patients, n** | 45 |
| Age, years, mean ± SD | 59.1 ± 11 |
| Gender, % (n) |  |
| Female | 37.8% (17) |
| Male | 62.2% (28) |
| Diabetes mellitus, % (n) | 20% (9) |
| Smoking habit |  |
| Nonsmoker/Former smoker | 75.6% (34) |
| ≤ 10 cigarettes/day | 6.7% (3) |
| ≥ 10 cigarettes/day | 17.8% (8) |

#### Supplementary Table 3. **Tooth-Level Clinical Data.**

| **Teeth, n** | 299 |
| --- | --- |
| Tooth arch, % (n) |  |
| Maxilla | 45.8% (137) |
| Mandible | 54.2% (162) |
| Tooth region, %, (n) |  |
| Incisor or canine | 35.1% (105) |
| Premolar | 31.4% (94) |
| Molar | 33.4% (100) |
| Root anatomy, %, (n) |  |
| Single-rooted | 56.9% (170) |
| Multi-rooted | 43.1% (129) |
| PD (mm) |  |
| Mean ± SD | 5 ± 1.8 |
| Median [IQR] | 5 [3; 6] |
| Minimum-Maximum | 2-14 |
| Furcation involvement, % (n) | (n = 129) |
| No | 52.7% (68) |
| Grade I | 19.4% (25) |
| Grade II | 27.1% (35) |
| Grade III | 0.8% (1) |
| Mobility, % (n) |  |
| No | 73.6% (220) |
| Grade I | 24.1% (72) |
| Grade II | 2.3% (7) |
| Grade III | 0% (0) |
| RBL, % (n) |  |
| <25% | 39.5% (118) |
| 25-50% | 44.1% (132) |
| >50% | 16.4% (49) |
| Apical pathology, % (n) |  |
| Normal periapical bone structure | 96% (287) |
| Small changes in periapical structure with no bone demineralization | 3% (9) |
| Periapical changes with diffuse mineral loss | 0.7% (2) |
| Severe periapical periodontitis | 0.3% (1) |
| Interproximal restoration (y/n) | 68/231 |
| Retainer (y/n) | 24/275 |
| RCT (y/n) | 17/282 |
| Vertical defect > 3mm (y/n) | 19/280 |

*IQR: interquartile range; PD: probing depth; RBL: radiographic bone loss; RCT: root canal treatment; SD: standard deviation.*

#### Supplementary Figure 2. Distribution of tooth loss causes among those that were lost.

## 3.2. Clinicians’ evaluation and Artificial intelligence

Individual responses from Periodontists, General Dentists and AI are shown in Supplementary Tables 4 to 6.

#### Supplementary Table 4. Periodontists: Score for Survival (0–10).

| **Evaluator** | **N** | **Mean** | **SD** | **Min** | **Max** | **P25** | **Median** | **P75** |
| --- | --- | --- | --- | --- | --- | --- | --- | --- |
| Perio 1 | 299 | 8.9 | 1.6 | 2.0 | 10.0 | 8.0 | 10.0 | 10.0 |
| Perio 2 | 299 | 8.2 | 2.3 | 0.0 | 10.0 | 7.0 | 9.0 | 10.0 |
| Perio 3 | 299 | 7.9 | 1.1 | 3.0 | 9.0 | 7.0 | 8.0 | 9.0 |
| Perio 4 | 299 | 8.3 | 1.5 | 2.0 | 10.0 | 7.0 | 8.0 | 9.0 |
| Perio 5 | 299 | 7.2 | 2.1 | 0.0 | 10.0 | 6.0 | 8.0 | 9.0 |
| Perio 6 | 299 | 6.7 | 2.4 | 1.0 | 10.0 | 5.0 | 7.0 | 9.0 |
| Perio 7 | 299 | 8.3 | 2.2 | 1.0 | 10.0 | 8.0 | 9.0 | 10.0 |
| Perio 8 | 299 | 6.3 | 2.2 | 0.0 | 10.0 | 5.0 | 7.0 | 8.0 |
| Perio 9 | 299 | 7.7 | 2.4 | 0.0 | 10.0 | 6.0 | 9.0 | 10.0 |
| Perio 10 | 299 | 9.5 | 1.2 | 0.0 | 10.0 | 9.0 | 10.0 | 10.0 |
| Perio 11 | 299 | 7.2 | 2.2 | 0.0 | 10.0 | 6.0 | 8.0 | 9.0 |
| Perio 12 | 299 | 7.3 | 2.1 | 1.0 | 10.0 | 6.0 | 8.0 | 9.0 |
| **Mean** | 299 | 7.8 | 1.6 | 1.8 | 9.9 | 6.8 | 8.3 | 9.1 |

*Perio = Periodontist; SD = Standard Deviation; P25 = 25th Percentile; P75 = 75th Percentile.*

#### Supplementary Table 5. General Dentists: Score for Survival (0–10).

| **Evaluator** | **N** | **Mean** | **SD** | **Min** | **Max** | **P25** | **Median** | **P75** |
| --- | --- | --- | --- | --- | --- | --- | --- | --- |
| GD 1 | 299 | 6.5 | 2.3 | 1.0 | 10.0 | 5.0 | 7.0 | 8.0 |
| GD 2 | 299 | 7.5 | 1.8 | 1.0 | 10.0 | 6.0 | 7.0 | 9.0 |
| GD 3 | 299 | 6.5 | 1.8 | 0.0 | 10.0 | 6.0 | 7.0 | 8.0 |
| GD 4 | 299 | 8.1 | 2.0 | 1.0 | 10.0 | 7.0 | 8.0 | 10.0 |
| GD 5 | 299 | 7.5 | 3.0 | 0.0 | 10.0 | 5.0 | 10.0 | 10.0 |
| GD 6 | 299 | 7.9 | 2.0 | 1.0 | 10.0 | 7.0 | 8.0 | 9.0 |
| GD 7 | 299 | 7.7 | 2.6 | 0.0 | 10.0 | 6.0 | 9.0 | 10.0 |
| GD 8 | 299 | 6.9 | 1.9 | 1.0 | 10.0 | 6.0 | 7.0 | 8.0 |
| GD 9 | 299 | 6.3 | 2.2 | 1.0 | 10.0 | 5.0 | 6.0 | 8.0 |
| GD 10 | 299 | 6.7 | 2.0 | 1.0 | 10.0 | 5.0 | 7.0 | 8.0 |
| GD 11 | 299 | 8.3 | 2.0 | 1.0 | 10.0 | 8.0 | 9.0 | 10.0 |
| **Mean** | 299 | 7.3 | 1.6 | 1.9 | 9.9 | 6.4 | 7.5 | 8.6 |

*GD = General Dentist; SD = Standard Deviation; P25 = 25th Percentile; P75 = 75th Percentile.*

#### Supplementary Table 6. AI: Score for Survival (0–10).

| **N** | **Mean** | **SD** | **Min** | **Max** | **P25** | **Median** | **P75** |
| --- | --- | --- | --- | --- | --- | --- | --- |
| 299 | 8.3 | 2.8 | 0 | 10 | 8 | 10 | 10 |

*SD = Standard Deviation; P25 = 25th Percentile; P75 = 75th Percentile.*

## 3.3. ROC curve analysis

#### Supplementary Figure 3. ROC curve analysis comparing the diagnostic performance of periodontists, GDs, and the AI model. **a.** For all-cause tooth loss, periodontists (AUC = 0.714 [CI 0.652-0.776]) and GDs (AUC = 0.711 [CI 0.648-0.774]) significantly outperformed the AI model (AUC = 0.621 [CI 0.550-0.693]; p<0.01 and p<0.05, respectively), with no significant difference between clinician groups. **b.** When limited to teeth lost due to periodontitis, AUC values were comparable across all groups (periodontists = 0.768 [CI 0.687-0.849], GDs = 0.751 [CI 0.665-0.837], AI = 0.751 [CI 0.681-0.820]; p>0.05).

#### Supplementary Table 7. ROC Analysis for AI, periodontists and general dentists.

|  | **AUC** | |
| --- | --- | --- |
|  | ***Tooth loss any causes*** | ***Tooth loss by periodontitis*** |
| **AI** | 0.621 (0.550-0.693) | 0.751 (0.681-0.820) |
| **PERIODONTISTS** | 0.714 (0.652-0.776) | 0.768 (0.687-0.849) |
| **GENERAL DENTISTS** | 0.711 (0.648-0.774) | 0.751 (0.665-0.837) |

#### Supplementary Table 8. Pairwise DeLong Test for AUC Comparison between AI, periodontists, and general dentists predicting tooth loss due to any causes or due to periodontitis.

|  | **p-value** | |
| --- | --- | --- |
|  | ***Tooth loss any causes*** | ***Tooth loss by periodontitis*** |
| **AI x PERIODONTISTS** | **0.008**** | 0.717 |
| **AI x GENERAL DENTISTS** | **0.015*** | 0.990 |
| **PERIODONTISTS x GENERAL DENTISTS** | 0.845 | 0.280 |

## 3.4. Analysis using a fixed threshold

Predictions of tooth loss and survival were dichotomized at the midpoint of the 0–10 scale (>5 = survival, ≤5 = loss), as shown in Figure 1. For example, a clinician giving a tooth a score of 6 (uncertain, leaning toward survival) is classified as “survival”.

Individual responses from Periodontists, General Dentists and AI are shown in Supplementary Tables 9 to 11.

#### Supplementary Table 9. Periodontists: Predicted Tooth Survival vs. Loss (Teeth with score > 5 were considered predictions as ‘saved’; Teeth with score ≤ 5 were considered predictions as ‘lost’).

| **Evaluator** | **N** | **Total %** | **Saved N** | **Saved %** | **Lost N** | **Lost %** |
| --- | --- | --- | --- | --- | --- | --- |
| Perio 1 | 299 | 100.0 | 283 | 94.6 | 16 | 5.4 |
| Perio 2 | 299 | 100.0 | 267 | 89.3 | 32 | 10.7 |
| Perio 3 | 299 | 100.0 | 290 | 97.0 | 9 | 3.0 |
| Perio 4 | 299 | 100.0 | 286 | 95.7 | 13 | 4.3 |
| Perio 5 | 299 | 100.0 | 238 | 79.6 | 61 | 20.4 |
| Perio 6 | 299 | 100.0 | 207 | 69.2 | 92 | 30.8 |
| Perio 7 | 299 | 100.0 | 261 | 87.3 | 38 | 12.7 |
| Perio 8 | 299 | 100.0 | 211 | 70.6 | 88 | 29.4 |
| Perio 9 | 299 | 100.0 | 227 | 75.9 | 72 | 24.1 |
| Perio 10 | 299 | 100.0 | 293 | 98.0 | 6 | 2.0 |
| Perio 11 | 299 | 100.0 | 231 | 77.3 | 68 | 22.7 |
| Perio 12 | 299 | 100.0 | 242 | 80.9 | 57 | 19.1 |
| **Mean** | 299 | 100.0 | 279 | **93.3** | 20 | **6.7** |

#### Supplementary Table 10. General Dentists: Predicted Tooth Survival vs. Loss (Teeth with score > 5 were considered predictions as ‘saved’; Teeth with score ≤ 5 were considered predictions as ‘lost’).

| **Evaluator** | **Total N** | **Total %** | **Saved N** | **Saved %** | **Lost N** | **Lost %** |
| --- | --- | --- | --- | --- | --- | --- |
| GD 1 | 299 | 100.0 | 208 | 69.6 | 91 | 30.4 |
| GD 2 | 299 | 100.0 | 257 | 86.0 | 42 | 14.0 |
| GD 3 | 299 | 100.0 | 229 | 76.6 | 70 | 23.4 |
| GD 4 | 299 | 100.0 | 270 | 90.3 | 29 | 9.7 |
| GD 5 | 299 | 100.0 | 172 | 57.5 | 127 | 42.5 |
| GD 6 | 299 | 100.0 | 258 | 86.3 | 41 | 13.7 |
| GD 7 | 299 | 100.0 | 229 | 76.6 | 70 | 23.4 |
| GD 8 | 299 | 100.0 | 235 | 78.6 | 64 | 21.4 |
| GD 9 | 299 | 100.0 | 184 | 61.5 | 115 | 38.5 |
| GD 10 | 299 | 100.0 | 217 | 72.6 | 82 | 27.4 |
| GD 11 | 299 | 100.0 | 271 | 90.6 | 28 | 9.4 |
| **Mean** | 299 | 100.0 | 265 | **88.6** | 34 | **11.4** |

#### Supplementary Table 11. AI: Predicted Tooth Survival vs. Loss (Teeth with score > 5 were considered predictions as ‘saved’; Teeth with score ≤ 5 were considered predictions as ‘lost’).

| **N** | **Total %** | **Saved N** | **Saved %** | **Lost N** | **Lost %** |
| --- | --- | --- | --- | --- | --- |
| 299 | 100.0 | 250 | **83.6** | 49 | **16.4** |

### 3.4.1. Prediction of tooth loss for any cause (periodontitis and other reasons)

Comparison of accuracy, sensitivity and specificity between AI, periodontists, and general dentists, as well as individual analysis, is provided in Supplementary Tables 12, 13 and 14.

#### Supplementary Table 12. Comparison of accuracy, sensitivity and specificity between AI, periodontists, and general dentists: McNemar’s test Chi^2^, degrees of freedom, and p-values for pairwise comparisons.

|  | **Accuracy** | | | **Sensitivity** | | | **Specificity** | | |
| --- | --- | --- | --- | --- | --- | --- | --- | --- | --- |
|  | **McNemar’s Chi^2^** | **df** | **p-value** | **McNemar’s Chi^2^** | **df** | **p-value** | **McNemar’s Chi^2^** | **df** | **p-value** |
| **AI vs Periodontists** | 5.89 | 1 | 0.015* | 0.84 | 1 | 0.359 | 14.69 | 1 | <0.001*** |
| **AI vs General Dentists** | 5.22 | 1 | 0.022* | 0.00 | 1 | 1 | 7.03 | 1 | 0.008** |
| **General Dentists vs Periodontists** | 0.05 | 1 | 0.823 | 3.13 | 1 | 0.077 | 4.08 | 1 | 0.043* |

#### Supplementary Table 13. Comparison of accuracy between AI and individual periodontists: McNemar’s test Chi^2^, degrees of freedom, and p-values.

|  | **McNemar’s Chi^2^** | **df** | **p-value** |
| --- | --- | --- | --- |
| **AI vs Perio1** | 0.92 | 1 | 0.337 |
| **AI vs Perio2** | 3.35 | 1 | 0.067 |
| **AI vs Perio3** | 7.22 | 1 | **0.007**** |
| **AI vs Perio4** | 4.33 | 1 | **0.038*** |
| **AI vs Perio5** | 0.00 | 1 | 1.000 |
| **AI vs Perio6** | 0.48 | 1 | 0.488 |
| **AI vs Perio7** | 2.76 | 1 | 0.097 |
| **AI vs Perio8** | 0.00 | 1 | 1.000 |
| **AI vs Perio9** | 0.00 | 1 | 1.000 |
| **AI vs Perio10** | 7.27 | 1 | **0.007**** |
| **AI vs Perio11** | 3.05 | 1 | 0.807 |
| **AI vs Perio12** | 3.02 | 1 | 0.082 |

#### Supplementary Table 14. Comparison of accuracy between AI and individual general dentists: McNemar’s test Chi^2^, degrees of freedom, and p-values.

|  | **McNemar’s Chi^2^** | **df** | **p-value** |
| --- | --- | --- | --- |
| **AI vs GD1** | 0.12 | 1 | 0.728 |
| **AI vs GD2** | 0.00 | 1 | 1.000 |
| **AI vs GD3** | 0.46 | 1 | 0.500 |
| **AI vs GD4** | 2.91 | 1 | 0.088 |
| **AI vs GD5** | 5.48 | 1 | 0.019 |
| **AI vs GD6** | 3.63 | 1 | 0.057 |
| **AI vs GD7** | 1.59 | 1 | 0.207 |
| **AI vs GD8** | 1.87 | 1 | 0.171 |
| **AI vs GD9** | 1.91 | 1 | 0.167 |
| **AI vs GD10** | 0.23 | 1 | 0.630 |
| **AI vs GD11** | 7.53 | 1 | **0.006**** |

### 3.4.2. **Prediction** of **tooth loss due to periodontitis**

Comparison of accuracy, sensitivity and specificity between AI, periodontists, and general dentists, as well as individual analysis, is provided in Supplementary Tables 15, 16 and 17.

#### Supplementary Table 15. Comparison of accuracy, sensitivity and specificity between AI, periodontists, and general dentists: McNemar’s test Chi^2^, degrees of freedom, and p-values for pairwise comparisons.

|  | **Accuracy** | | | **Sensitivity** | | | | **Specificity** | | |
| --- | --- | --- | --- | --- | --- | --- | --- | --- | --- | --- |
|  | **McNemar’s Chi^2^** | **df** | **p-value** | **McNemar’s Chi^2^** | | **df** | **p-value** | **McNemar’s Chi^2^** | **df** | **p-value** |
| **AI vs Periodontists** | 10.5 | 1 | **0.001**** | | 0.07 | 1 | 0.789 | 16.5 | 1 | **<0.001***** |
| **AI vs General Dentists** | 6.61 | 1 | **0.010*** | | 0.08 | 1 | 0.773 | 6.92 | 1 | **0.009**** |
| **General Dentists vs Periodontists** | 1.25 | 1 | 0.264 | | 2.25 | 1 | 0.134 | 5.06 | 1 | **0.024*** |

#### *Supplementary Table 16.* Comparison of accuracy between AI and individual periodontists: McNemar’s test Chi^2^, degrees of freedom, and p-values.

|  | **McNemar’s Chi^2^** | **df** | **p-value** |
| --- | --- | --- | --- |
| **AI vs Perio1** | 5.03 | 1 | **0.025*** |
| **AI vs Perio2** | 4.56 | 1 | **0.033*** |
| **AI vs Perio3** | 10.58 | 1 | **0.001**** |
| **AI vs Perio4** | 8.48 | 1 | **0.004**** |
| **AI vs Perio5** | 0.00 | 1 | 1.000 |
| **AI vs Perio6** | 2.61 | 1 | 0.106 |
| **AI vs Perio7** | 3.61 | 1 | 0.058 |
| **AI vs Perio8** | 4.06 | 1 | **0.044*** |
| **AI vs Perio9** | 0.00 | 1 | 1.000 |
| **AI vs Perio10** | 12.29 | 1 | **<0.001***** |
| **AI vs Perio11** | 0.96 | 1 | 0.328 |
| **AI vs Perio12** | 0.00 | 1 | 1.000 |

#### Supplementary Table 17. Comparison of accuracy between AI and individual general dentists: McNemar’s test Chi^2^, degrees of freedom, and p-values.

|  | **McNemar’s Chi^2^** | **df** | **p-value** |
| --- | --- | --- | --- |
| **AI vs GD1** | 8.45 | 1 | **0.004**** |
| **AI vs GD2** | 0.43 | 1 | 0.511 |
| **AI vs GD3** | 0.20 | 1 | 0.653 |
| **AI vs GD4** | 0.84 | 1 | 0.358 |
| **AI vs GD5** | 22.82 | 1 | **<0.001***** |
| **AI vs GD6** | 1.31 | 1 | 0.253 |
| **AI vs GD7** | 1.02 | 1 | 0.313 |
| **AI vs GD8** | 0.00 | 1 | 1.000 |
| **AI vs GD9** | 20.35 | 1 | **<0.001***** |
| **AI vs GD10** | 3.71 | 1 | 0.054 |
| **AI vs GD11** | 5.95 | 1 | **0.015*** |

## 3.5. Analysis using group-specific optimal thresholds

Instead of applying the same midpoint cutoff for all evaluators, this approach identifies the threshold that best balances sensitivity and specificity using Youden’s Index. For the AI model, the optimal decision threshold was adopted from the original development and validation study (Santamaria et al., 2024). For the human control groups, optimized thresholds were derived within a 10-fold cross-validation framework, with each fold serving as a quasi-validation set prior to performance evaluation. For tooth loss for any cause, optimal classification thresholds were 8.9 for periodontists, 7.1 for general dentists, and 7 for AI (Figure 1). For tooth loss due to periodontitis, the corresponding optimal thresholds were 6.3 for periodontists, 6.1 for general dentists, and 7 for AI.

***3.5.2. Prediction of tooth loss for any cause***

Individual responses from Periodontists, General Dentists and AI are shown in Supplementary Tables 18 to 20.

#### Supplementary Table 18. Periodontists: Predicted Tooth Survival vs. Loss (Teeth with score > 8.9 were considered predictions as ‘saved’; Teeth with score ≤ 8.9 were considered predictions as ‘lost’).

| **Evaluator** | **N** | **Total %** | **Saved N** | **Saved %** | **Lost N** | **Lost %** |
| --- | --- | --- | --- | --- | --- | --- |
| Perio 1 | 299 | 100.0 | 156 | 52.2 | 143 | 47.8 |
| Perio 2 | 299 | 100.0 | 169 | 56.5 | 130 | 43.5 |
| Perio 3 | 299 | 100.0 | 214 | 71.6 | 85 | 28.4 |
| Perio 4 | 299 | 100.0 | 70 | 23.4 | 229 | 76.6 |
| Perio 5 | 299 | 100.0 | 119 | 39.8 | 180 | 60.2 |
| Perio 6 | 299 | 100.0 | 136 | 45.5 | 163 | 54.5 |
| Perio 7 | 299 | 100.0 | 148 | 49.5 | 151 | 50.5 |
| Perio 8 | 299 | 100.0 | 162 | 54.2 | 137 | 45.8 |
| Perio 9 | 299 | 100.0 | 181 | 60.5 | 118 | 39.5 |
| Perio 10 | 299 | 100.0 | 214 | 71.6 | 85 | 28.4 |
| Perio 11 | 299 | 100.0 | 140 | 46.8 | 159 | 53.2 |
| Perio 12 | 299 | 100.0 | 193 | 64.5 | 106 | 35.5 |
| **Mean** | 299 | **100.0** | 99 | **33.1** | 200 | **66.9** |

#### Supplementary Table 19. General Dentists: Predicted Tooth Survival vs. Loss (Teeth with score > 7.1 were considered predictions as ‘saved’; Teeth with score ≤ 7.1 were considered predictions as ‘lost’).

| **Evaluator** | **Total N** | **Total %** | **Saved N** | **Saved %** | **Lost N** | **Lost %** |
| --- | --- | --- | --- | --- | --- | --- |
| GD 1 | 299 | 100.0 | 167 | 55.9 | 132 | 44.1 |
| GD 2 | 299 | 100.0 | 105 | 35.1 | 194 | 64.9 |
| GD 3 | 299 | 100.0 | 177 | 59.2 | 122 | 40.8 |
| GD 4 | 299 | 100.0 | 205 | 68.6 | 94 | 31.4 |
| GD 5 | 299 | 100.0 | 172 | 57.5 | 127 | 42.5 |
| GD 6 | 299 | 100.0 | 209 | 69.9 | 90 | 30.1 |
| GD 7 | 299 | 100.0 | 152 | 50.8 | 147 | 49.2 |
| GD 8 | 299 | 100.0 | 151 | 50.5 | 148 | 49.5 |
| GD 9 | 299 | 100.0 | 248 | 82.9 | 51 | 17.1 |
| GD 10 | 299 | 100.0 | 201 | 67.2 | 98 | 32.8 |
| GD 11 | 299 | 100.0 | 194 | 64.9 | 105 | 35.1 |
| **Mean** | 299 | **100.0** | 142 | **47.5** | 157 | **52.5** |

#### Supplementary Table 20. AI: Predicted Tooth Survival vs. Loss (Teeth with score > 7 were considered predictions as ‘saved’; Teeth with score ≤ 7 were considered predictions as ‘lost’).

| **N** | **Total %** | **Saved N** | **Saved %** | **Lost N** | **Lost %** |
| --- | --- | --- | --- | --- | --- |
| 299 | 100.0 | 235 | **78.6** | 64 | **21.4** |

Comparison of accuracy, sensitivity and specificity between AI, periodontists, and general dentists, as well as individual analysis, is provided in Supplementary Tables 21, 22 and 23.

#### Supplementary Table 21. Comparison of accuracy, sensitivity and specificity between AI, periodontists, and general dentists: p-values for pairwise comparisons using McNemar’s test.

|  | **Accuracy** | **Sensitivity** | **Specificity** |
| --- | --- | --- | --- |
| **AI vs Periodontists** | **0.003**** | **<0.001***** | **<0.001***** |
| **AI vs General Dentists** | **0.004**** | **<0.001***** | **<0.001***** |
| **General Dentists vs Periodontists** | 0.597 | **<0.001***** | **<0.001***** |

#### *Supplementary Table 22.* Comparison of accuracy between AI and individual periodontists: p-values for McNemar’s test.

|  | **p-value** |
| --- | --- |
| **AI vs Perio1** | **0.040*** |
| **AI vs Perio2** | 0.050 |
| **AI vs Perio3** | 1 |
| **AI vs Perio4** | **<0.001***** |
| **AI vs Perio5** | **<0.001***** |
| **AI vs Perio6** | **<0.001***** |
| **AI vs Perio7** | **0.002**** |
| **AI vs Perio8** | 0.407 |
| **AI vs Perio9** | 0.572 |
| **AI vs Perio10** | 0.489 |
| **AI vs Perio11** | **0.004**** |
| **AI vs Perio12** | 0.908 |

#### Supplementary Table 23. Comparison of accuracy between AI and individual general dentists: p-values for McNemar’s test.

|  | **p-value** |
| --- | --- |
| **AI vs GD1** | 0.251 |
| **AI vs GD2** | **<0.001***** |
| **AI vs GD3** | **0.041*** |
| **AI vs GD4** | 0.624 |
| **AI vs GD5** | 0.057 |
| **AI vs GD6** | 0.451 |
| **AI vs GD7** | **0.015*** |
| **AI vs GD8** | **0.008**** |
| **AI vs GD9** | 0.142 |
| **AI vs GD10** | 0.728 |
| **AI vs GD11** | 0.228 |

*3.5.3.* ***Prediction of*** ***tooth loss due to periodontitis***

Comparison of accuracy, sensitivity and specificity between AI, periodontists, and general dentists, as well as individual analysis, is provided in Supplementary Tables 24, 25 and 26.

#### Supplementary Table 24. Comparison of accuracy, sensitivity and specificity between AI, periodontists, and general dentists: p-values for pairwise comparisons using McNemar’s test.

|  | **Accuracy** | **Sensitivity** | **Specificity** |
| --- | --- | --- | --- |
| **AI vs Periodontists** | **<0.001***** | 0.804 | **<0.001***** |
| **AI vs General Dentists** | 1 | 0.332 | 0.551 |
| **General Dentists vs Periodontists** | **<0.001***** | 0.375 | **<0.001***** |

#### *Supplementary Table 25.* Comparison of accuracy between AI and individual periodontists: p-values for McNemar’s test.

|  | **p-value** |
| --- | --- |
| **AI vs Perio1** | **<0.001***** |
| **AI vs Perio2** | 0.341 |
| **AI vs Perio3** | **<0.001***** |
| **AI vs Perio4** | 1 |
| **AI vs Perio5** | **<0.001***** |
| **AI vs Perio6** | **0.012*** |
| **AI vs Perio7** | 0.160 |
| **AI vs Perio8** | **<0.001***** |
| **AI vs Perio9** | 0.901 |
| **AI vs Perio10** | 0.356 |
| **AI vs Perio11** | 0.561 |
| **AI vs Perio12** | **0.007**** |

#### Supplementary Table 26. Comparison of accuracy between AI and individual general dentists: p-values using McNemar’s test.

|  | **p-value** |
| --- | --- |
| **AI vs GD1** | **<0.001***** |
| **AI vs GD2** | **0.002**** |
| **AI vs GD3** | 0.142 |
| **AI vs GD4** | **0.012*** |
| **AI vs GD5** | **<0.001***** |
| **AI vs GD6** | 0.451 |
| **AI vs GD7** | **<0.001***** |
| **AI vs GD8** | 0.434 |
| **AI vs GD9** | **<0.001***** |
| **AI vs GD10** | 0.082 |
| **AI vs GD11** | **<0.001***** |

## 3.6. Calibration performance

Calibration of the AI model, experienced periodontists, and general dentists was assessed using the Brier score, the observed/expected (O/E) ratio, and the calibration intercept and slope. The AI model showed a calibration slope of 0.12, an intercept of 1.03, and an O/E ratio of 1.44, indicating substantial miscalibration and systematic overestimation of tooth survival probability. Experienced periodontists showed a smaller deviation from ideal calibration (slope 0.82, intercept 0.19, O/E ratio 1.13), while the mean predictions of general dentists were closely calibrated (slope 0.94, intercept -0.14, O/E ratio 0.91). Brier scores were 0.247 for the AI model, versus 0.171 and 0.170 for periodontists and general dentists, respectively.

Supplementary Table 27. Calibration performance of the AI model and human raters for 10-year tooth loss prediction.

| **Predictor** | **Brier score** | **O/E ratio** | **Calibration intercept** | **Calibration slope** |
| --- | --- | --- | --- | --- |
| AI model | 0.247 | 1.44 | 1.03 | 0.12 |
| Experienced periodontists (mean) | 0.171 | 1.13 | 0.19 | 0.82 |
| General dentists (mean) | 0.170 | 0.91 | −0.14 | 0.94 |

*O/E ratio: Observed/Expected ratio*

**References**

Santamaria, P., Troiano, G., Serroni, M., Araùjo, T. G., Ravidà, A., & Nibali, L. (2024). Exploring the accuracy of tooth loss prediction between a clinical periodontal prognostic system and a machine learning prognostic model. *J Clin Periodontol, 51*(10), 1333-1341. doi:10.1111/jcpe.14023
